# Supplementary material for: The causal role of circulating inflammatory markers in osteoporosis: a bidirectional Mendelian randomized study
Source: Front Immunol. 2024 Jul 18;15:1412298. doi: 10.3389/fimmu.2024.1412298 (PMC11291241; doi:10.3389/fimmu.2024.1412298)
Supplement: Supplementary file 1 [file DataSheet_1.zip › supplementary material/CIM-OSTEOPOROSIS /GCST90274835.csv_leave_one_out.pdf]

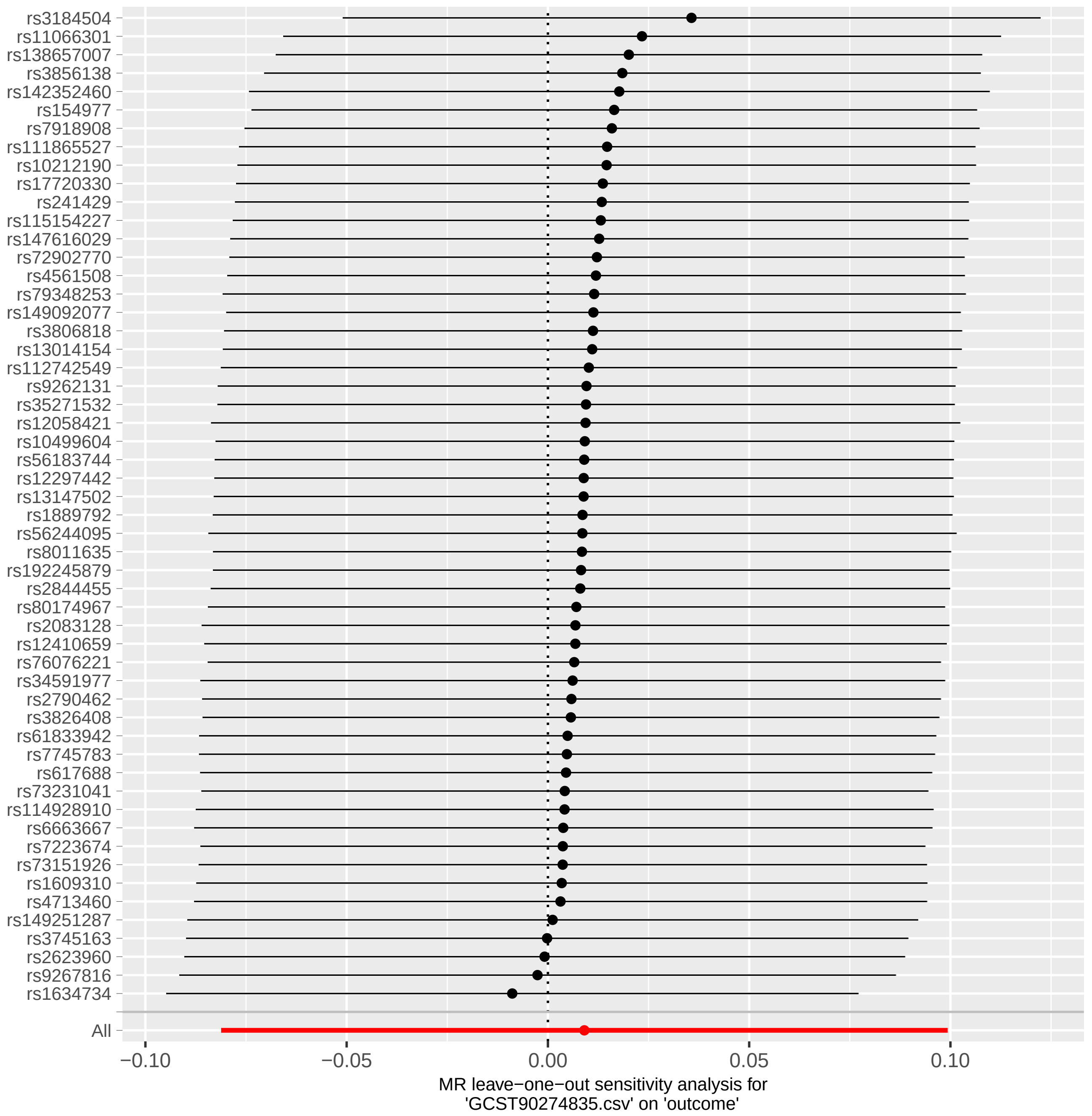
rs3184504
rs11066301
rs138657007
rs3856138
rs142352460
rs154977
rs7918908
rs111865527
rs10212190
rs17720330
rs241429
rs115154227
rs147616029
rs72902770
rs4561508
rs79348253
rs149092077
rs3806818
rs13014154
rs112742549
rs9262131
rs35271532
rs12058421
rs10499604
rs56183744
rs12297442
rs13147502
rs1889792
rs56244095
rs8011635
rs192245879
rs2844455
rs80174967
rs2083128
rs12410659
rs76076221
rs34591977
rs2790462
rs3826408
rs61833942
rs7745783
rs617688
rs73231041
rs114928910
rs6663667
rs7223674
rs73151926
rs1609310
rs4713460
rs149251287
rs3745163
rs2623960
rs9267816
rs1634734
All
−0.10
−0.05
0.00
0.05
0.10
MR leave−one−out sensitivity analysis for
'GCST90274835.csv' on 'outcome'
